# Antiretroviral treatment is less effective at reducing gut microbiome-associated inflammation and T cell activation in people living with HIV in rural versus urban Zimbabwe

**DOI:** 10.21203/rs.3.rs-3300723/v1

**Published:** 2023-08-31

**Authors:** Alessandro Lazzaro, Angela Sofia Burkhart Colorado, Charles Preston Neff, Nichole Nusbacher, Kathryn Boyd, Suzanne Fiorillo, Casey Martin, Janet Siebert, Thomas Campbell, Margaret Borok, Brent Palmer, Catherine Lozupone

**Affiliations:** Sapienza University of Rome; University of Colorado Anschutz Medical Campus; University of Colorado Anschutz Medical Campus; University of Colorado Anschutz Medical Campus; London School of Hygiene & Tropical Medicine; University of Colorado Anschutz Medical Campus; University of Colorado Anschutz Medical Campus; University of Colorado Anschutz Medical Campus; University of Colorado Anschutz Medical Campus; University of Zimbabwe; University of Colorado Anschutz Medical Campus; University of Colorado Anschutz Medical Campus

**Keywords:** HIV, intestinal microbiome, ART response, immune activation and exhaustion, inflammation, urban, rural

## Abstract

The widespread availability of antiretroviral therapy (ART) for people living with HIV (PLWH) has dramatically reduced mortality and improved life expectancy. However, even with suppression of HIV-1 replication, chronic immune activation and elevated inflammation persist. Chronic immune activation has been linked to a pro-inflammatory gut microbiome composition, exacerbated by compromised intestinal barrier integrity that occurs after HIV infection. Individuals living in urban versus rural areas of sub-Saharan Africa have differences in environmental factors such as water source or diet that may impact gut microbiome composition, yet immune phenotype and gut microbiome composition response to ART in PLWH living in rural versus urban areas of sub-Saharan Africa have not been compared. Here, we measured immune phenotypes and fecal microbiome composition in PLWH and healthy participants recruited from the urban Mabvuku polyclinic in the city of Harare, Zimbabwe and the Mutoko District hospital located in a district 146 km from Harare that services surrounding rural villages. PLWH were either ART naïve at baseline and sampled again after 24 weeks of treatment with efavirenz/lamivudine/tenofovir disoproxil fumarate (EFV/3TC/TDF) and the prophylactic antibiotic cotrimoxazole or were ART experienced at both timepoints. Although expected reductions in the inflammatory marker IL-6, T-cell activation, and exhaustion were observed in individuals who had suppressed HIV-1 with treatment, these changes were significant only when considering individuals in the urban and not the rural area. Gut microbiome composition showed more marked differences from healthy controls in the ART experienced compared to ART naïve cohort, and consistent longitudinal changes were also observed in ART naïve PLWH after 24 weeks of treatment, including a reduction in alpha diversity and altered composition. However, gut microbiome composition showed a more pronounced relationship with chronic immune activation and exhaustion phenotypes in the ART naïve compared to ART experienced PLWH, suggesting a particularly significant role for the gut microbiome in disease progression in uncontrolled infection.

## Introduction

Human Immunodeficiency Virus 1 (HIV-1) infection is characterized by progressive infection and depletion of CD4 + T cells, chronic immune activation, and immune exhaustion that predisposes the infected individual to opportunistic infections and cancers defining acquired immunodeficiency syndrome (AIDS). Antiretroviral therapy (ART) has dramatically improved health outcomes in people living with HIV infection (PLWH). However, ART coverage remains suboptimal in many parts of the developing world, including in sub-Saharan Africa (SSA) where about 70% of the global HIV epidemic is concentrated [1], and is particularly challenging in rural areas [2]. Even with successful ART, people living with PLWH often have chronic immune activation and elevated inflammation [3] that has been linked with poor CD4 + T cell recovery [4] and the premature onset of HIV-related non-AIDS-defining comorbidities [5]. Understanding factors related to chronic immune activation with ART is essential for devising strategies to protect the health of PLWH.

Factors that can drive chronic immune activation in PLWH on effective ART include co-infections and persistent antigen stimulation from residual viremia or the intestinal microbiome [5]. This complex community of microbes has been of intense interest in HIV-infected populations, in part because HIV-1 disrupts gut associated lymphoid tissue (GALT) causing gut mucosa damage that allows for the translocation of inflammatory bacterial components [6]. Studies conducted in the US and Europe have found gut microbiome differences in HIV-infected populations that could be a result of HIV-driven immune dysfunction, ART drugs, or lifestyle factors [7–9]. These altered microbiomes have been shown to correlate with chronic T cell activation *in vivo* and to drive higher T cell activation *in vitro* [10] and cytokine production *ex vivo* [11]. Relatively few studies have investigated effects of HIV-1 or ART on the gut microbiome in SSA [12–15], and yet knowledge from the developed world may not be generalizable for several reasons. First, there are dramatic differences in gut microbiome composition in healthy individuals in the developing versus the developed world that are associated with differences in diet and environmental factors [16]. Second, the predominant mode of HIV-1 transmission differs in SSA compared to other regions, and related differences in demographic factors and behaviors influence gut microbiome composition [17]. Finally, concomitant use of the antibiotic cotrimoxazole to prevent opportunistic infections with ART regardless of CD4 + T cell count is more common in SSA compared to developed countries [18].

Individuals living near urban centers versus in rural areas of sub-Saharan Africa have differences in environmental exposures such as water source or diet that may impact their gut microbiome composition and immune response to microbes [19, 20]. There may also be different responses to ART in rural areas, although studies generally have focused on measuring rates of treatment failure rather than on differences in immune response following virologic control [21, 22]. To gain a further understanding of effects of HIV treatment on the gut microbiome and immune activation and inflammation in PLWH in SSA, and whether living in a rural versus urban area can influence the effects ART, we designed a prospective longitudinal observational study of participants from rural and urban hospitals in Zimbabwe. We compared immune phenotypes and gut microbiome composition among ART naïve and ART experienced PLWH and healthy controls (HCs) and assessed effects of 24 weeks of ART and cotrimoxazole using longitudinal analysis.

## Results

### Demographic and Clinical Characteristics of Study Cohort

We recruited 162 individuals with approximately equal numbers from the urban Mabvuku Polyclinic in the city of Harare and the Mutoko District Hospital, located in a district of around 161,000 people [23] 146 km from Harare that services surrounding rural villages. Blood immune profile and fecal microbiome composition were evaluated in samples collected at two timepoints 24 weeks apart in 1) PLWH who were not on ART at the first timepoint but who subsequently commenced first-line ART with efavirenz/lamivudine/tenofovir disoproxil fumarate (EFV/3TC/TDF) and the prophylactic antibiotic cotrimoxazole (ART Naïve), 2) PLWH who were on this same ART regimen and cotrimoxazole at both timepoints (ART experienced), and 3) people without HIV, hereafter referred to as healthy controls (HC). Of the 162 enrolled individuals across the three cohorts, 14 from the ART naïve cohort were excluded because they had HIV viral load below 20 at the baseline visit, which is inconsistent with their declared treatment status (Figure S1). Ultimately, 148 individuals were analyzed with 67 individuals in the ART naïve cohort, 39 in the ART experienced cohort, and 42 HCs (Table 1). Furthermore, 15 individuals who provided samples at the baseline visit were lost to follow-up at the 24 week visit, so longitudinal analyses included 266 samples from 133 total individuals (Figure S1).

Clinical and demographic characteristics of study population by cohort at baseline visit. P-values were calculated using the Mann Whitney U test. Virologic failure is defined as PLWH who are on ART but have uncontrolled viral replication(> 200 copies of HIV-1 RNA/mL of plasma) [24, 25]. BMI categories were determined using World Health Organization (WHO) standards [26]. P-values are coded as ‘****’ between [0, 0.0001], ‘***’ (0.0001, 0.001], ‘**’ (0.001, 0.01], ‘*’ (0.01, 0.05], with square brackets indicating that the endpoints are included in the interval. NA represents values not collected/relevant to a particular cohort. Values are reported as the median with the interquartile range in brackets.

Cohorts had similar proportions of females versus males (overall 57% female). ART naïve PLWH and HC had a similar median age of 35 and 36 respectively and ART experienced PLWH were significantly older (median age of 45). Median Body Mass Index (BMI) was in the normal range for the overall population (BMI = 22) and HCs had higher BMI compared to both ART Naïve and experienced PLWH in the rural (24 versus 22) but not the urban area (Table S1). Women had a significantly higher BMI than men, with more men (~ 11–17%) than women (~ 2%) in the underweight categories (Table S2). The median duration of ART and cotrimoxazole treatment among the ART experienced cohort at baseline was 89 and 84 months respectively. The median duration of cotrimoxazole treatment at baseline was 1 day (interquartile range (IQR) 0–6.6 days) in the ART naïve cohort (Table 1), but significantly longer in the rural (~ 4 days; IQR 2.1–13.2) compared to the urban (~ 0 days; IQR 0–0) location (Table S1). PLWH recruited in the rural and urban locations did not have significantly different CD4 + T cell percent or CD4/CD8 T cell ratio at baseline (Table S1). ART naïve HIV + women had significantly higher CD4 + T cell percent and CD4/CD8 ratio compared to men (Table S2).

### Cross-sectional and Longitudinal Analysis of Immune Phenotype

As expected, the ART naïve cohort had higher HIV viral loads and lower CD4 + T cell count, CD4 + T cell percent, and CD4/CD8 T cell ratio compared to the ART experienced cohort at baseline (Table 1; Figure S2). All but 15 (22.4%) of the ART naïve individuals reached virologic control, defined as viral load < 200 copies of HIV-1/mL of plasma, after 24 weeks of therapy (Table 1) and 7 (17.9%) of the 39 individuals in the ART experienced cohort had uncontrolled HIV infection at baseline despite ART (Table 1). Levels of virologic control with ART were not significantly different between the urban and rural sites (Table S1). The individuals that remained viremic with treatment were removed from subsequent analyses. A significant increase in CD4/CD8 T cell ratio was observed after 24 weeks of treatment among individuals in the ART naïve cohort who reached virologic control in both the urban and rural sites (Figure S2).

We next used flow cytometry to gain a deeper understanding of the effects of successful ART on CD4 + and CD8 + T-cell populations in blood. The immune characterization included: 1) chronic immune activation using HLA-DR and CD38 as markers, a common measure of disease progression in studies of HIV [27–32]; 2) a marker of immune exhaustion, PD-1, which prior studies have shown to increase in PLWH [33–35]; and 3) mucosal trafficking by examination of CD103 expression, which can either increase in blood with challenge as populations expand, or decrease as they traffic from blood to mucosal sites [36, 37]. We also evaluated inflammation, by measuring plasma levels IL-6 and CRP using ELISA, both of which have been shown to increase in PLWH [38, 39].

### Immune Activation, Exhaustion, and Inflammation

**Figure 1:**
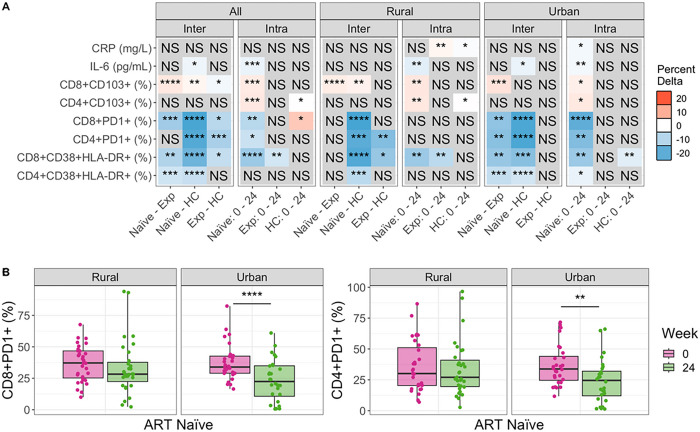
Differences in immune markers across cohorts and over time, before and after commencement of ART **(A)** Comparing immune marker values between cohorts (Inter) and across timepoints (Intra). Inter-cohort comparisons were performed on Baseline (week 0) samples using a Kruskal Wallis with a Dunn’s post-hoc test. Intra-cohort comparisons compared week 0 to week 24 using a paired Mann Whitney U test. Significant relationships are colored by the mean change in value to show directionality: For any given comparison (e.g., Naive – Exp), the box is colored red (up) if the average value of Exp is higher than that of Naive. If blue (down) the average value of Exp is lower than Naive. The opacity of the color indicates the strength of the change. Detailed plots for significant relationships are shown in Figure S4 (CD8+CD38+HLA-DR+ and CD4+CD38+HLA-DR+), Figure S5 (CD4+PD1+ and CD8+PD1+), Figure S6 (CD8+CD103+ and CD4+CD103+), and Figure S7 (IL-6 and CRP). **(B)** Stratified analysis of CD8 and CD4 PD1 percent change over time by location. P-values are coded as ‘****’ between [0, 0.0001], ‘***’ (0.0001, 0.001], ‘**’ (0.001, 0.01], ‘*’ (0.01, 0.05], with square brackets indicating that the endpoints are included in the interval. NS= not significant. Naïve=ART naive cohort, Exp=ART experienced cohort.

As expected, ART naïve PLWH had higher levels of chronically activated (CD38 + HLA-DR+) and exhausted (PD1+) CD4 + and CD8 + T cells compared to HC (Fig. 1, Figures S4, S5). Compared to ART naïve, PLWH who were ART experienced at baseline, had significantly lower levels of chronically activated CD4 + and CD8 + T cells and exhausted CD8 + PD1 + T cells (Fig. 1, Figures S4, S5), showing expected improvement with ART. However, when conducting the same analyses stratified by the urban versus rural location, significantly lower T cell activation and exhaustion levels in ART experienced versus ART naive PLWH were found only in the urban and not the rural location (Fig. 1, Figures S4, S5). ART experienced PLWH also had significantly higher levels of CD4 + and CD8 + T cell exhaustion compared to HCs, indicating incomplete recovery with ART, but statistically significant differences were observed in the rural and not urban cohort in stratified analysis (Fig. 1, Figures S4, S5). The inflammatory marker IL-6 also showed highest level at baseline in the ART naïve cohort, followed by the ART experienced cohort, while the HCs had the lowest levels. However, only a reduction in IL-6 between ART naïve PLWH and the HCs was statistically significant, and this difference was significant in the urban and not rural location in stratified analyses (Fig. 1A, Figure S7). Taken together, these results show ART experienced individuals to have expected improvements in inflammation and T cell activation and exhaustion compared to ART naïve PLWH in the urban but not the rural area. Accordingly, ART experienced individuals in the rural and not the urban area had significant differences from HC in immune activation and exhaustion phenotypes.

Longitudinal analysis of the ART naïve cohort showed consistent results. There was a significant reduction in CD4 + and CD8 + T cell exhaustion and CD8 + but not CD4 + chronic T cell activation with 24 weeks treatment overall (Fig. 1, Figures S4, S5). However, again this was driven by improvements only in the urban area; individuals from the urban area showed a significant reduction with 24 weeks of ART in all CD4 + and CD8 + T cell activation and exhaustion markers but the PLWH in the rural area showed a significant reduction in CD8 + T cell activation only (Fig. 1). ART naïve PLWH in the urban area had a significant reduction in both IL-6 and CRP following 24 weeks of ART, but in the rural area only IL-6 was reduced. Overall, these results suggest that the people living in the urban location exhibit better immune reconstitution with ART. This is the case even though we restricted our analyses to include only individuals with controlled viral replication and the rural and urban sites did not differ in the percent of individuals who achieved virologic control with ART.

One result suggesting a potential confounder is that the HCs exhibited increased CD4 + and CD8 + T cell exhaustion over time, even though no intervention occurred in this cohort. Similarly, the ART experienced cohort showed an increase in CRP over time in the rural and not the urban location (Fig. 1A). Changes over time without an intervention may be related to increased socio-economic stressors in Zimbabwe between January 2018 and August 2019 [40], when these samples were collected. To correct for these potential confounders, we also applied a linear modeling approach to determine whether the changes observed with treatment of the ART naïve cohort were greater than those observed over time in the HC cohort (Methods Model M1; Figure S3). These results suggested that improvements in CD4 + PD1 + and CD8 + PD1 + T cells with 24 weeks of ART were underestimated. They also showed that CRP results were impacted by confounding effects over time. When measured as change in the ART naïve cohort relative to the HCs, we found that CRP decreased with 24 weeks of ART in the urban area, as would be expected, but actually increased in the rural area (Figure S3).

### Mucosal Trafficking

One goal of this study was to relate immune markers in HIV to the intestinal microbiome. Thus, we were also interested in evaluating T cells expressing the mucosal trafficking marker CD103, since some of these cells might be trafficking to or from intestinal sites. CD8 + CD103 + T cells were highest in the ART experienced cohort and lowest in the ART naïve. CD4 + CD103 + T cells did not differ significantly across cohorts at baseline (Fig. 1, Figures S3, S7). Change over time in the ART naïve cohort showed that both CD4 + and CD8 + T cells expressing CD103 increased with treatment (Fig. 1A). The HC cohort showed a significant increase in mucosal trafficking over time in the rural area (Fig. 1A), but linear modeling confirmed that the change over time in ART naïve PLWH was still significantly increased relative to HC (Figure S3).

### Cross-sectional and Longitudinal Analysis of Microbiome Diversity

#### Alpha Diversity

**Figure 2:**
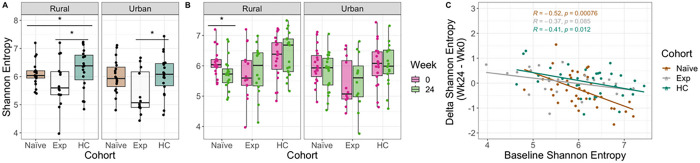
Alpha diversity across cohorts stratified by time and location. **(A)** Shannon Entropy differences between cohorts at baseline stratified by location. P-values were calculated using a Kruskal-Wallis test with a Dunn’s post hoc (pairwise comparisons shown). **(B)** Shannon entropy between time points for each cohort stratified by location. P-values were calculated using a paired Mann Whitney U test. **(C)** Linear regression of the difference in Shannon entropy (Shannon entropy at week 24 minus week 0) by baseline Shannon entropy (Shannon entropy at week 0). P-values are coded as ‘****’ between [0, 0.0001], ‘***’ (0.0001, 0.001], ‘**’ (0.001, 0.01], ‘*’ (0.01, 0.05], with square brackets indicating that the endpoints are included in the interval.

We next evaluated intestinal microbiome composition using 16S ribosomal RNA (rRNA)-targeted sequencing of fecal samples. One potential confounder in understanding the effects of ART on the microbiome in PLWH is the concomitant use of the antibiotic cotrimoxazole. There was also some exposure of ART naïve individuals to cotrimoxazole at baseline, and this was greater in people living in the rural versus urban location (Table 1). Despite this, ART naïve individuals in the rural area did not have significantly lower alpha diversity (measured with Shannon entropy [41]) at baseline (Mann Whitney U test, p > 0.05) and length of cotrimoxazole treatment at baseline did not correlate with alpha diversity (Figure S8). Individuals in the ART naïve cohort had reduced Shannon entropy compared to HCs in the rural and not the urban location (Fig. 2A).

The ART experienced cohort had the lowest alpha diversity at baseline in both locations (Fig. 2A). ART naive patients had a significant decrease in alpha diversity following 24 weeks of ART in the rural location only (Fig. 2B). The effect of 24 weeks of treatment on microbiome diversity was significantly related to baseline values (Fig. 2C, see Methods Model M2). Specifically, individuals with the highest baseline diversity showed the greatest loss, and those with the lowest baseline diversity on average showed improvement (positive change in alpha diversity), in both urban and rural areas.

#### Beta Diversity

**Figure 3:**
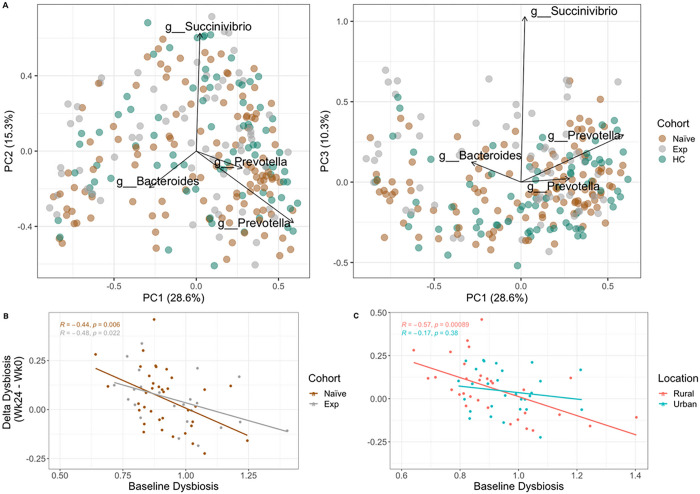
Biplots of Weighted UniFrac PCoA axes and delta dysbiosis analyses stratified by cohort and location. **(A)** Plotted weighted UniFrac PCoA axes that account for over 50% of the variance with important Amplicon Sequence Variants (ASVs) (importance defined as those farthest from the origin using Euclidean distance) indicated (Silva taxonomic assignments of important ASVs are indicated). **(B)** Linear regression model showing delta dysbiosis as a function of baseline dysbiosis stratified by cohort. Dysbiosis for each sample in ART naïve and experienced cohorts was calculated by taking the average weighted UniFrac distance from sample to healthy controls (HC). This average was calculated on values of the same timepoint. For example, if the sample had been taken at week 0, the dysbiosis score (average distance to HCs) was calculated with values only at week 0. Delta dysbiosis was calculated by taking the difference in dysbiosis between week 24 and week 0. **(C)** Same analysis as **B** but stratified by location.

Individuals in Zimbabwe had microbiome composition typical of those previously described in studies of SSA [42], with relatively high relative abundance of bacteria in the genus *Prevotella* (17.71% mean relative abundance +/− 13.07%) and low *Bacteroides* (9.42% mean relative abundance +/− 9.26%) (Figure S9). In a principal coordinate analysis (PCoA) of weighted UniFrac [43] values to visualize beta diversity, principal coordinate 1 (PC1) separated individuals by differences in Amplicon Sequence Variants (ASVs) assigned to the *Prevotella* and *Bacteroides* genera (Fig. 3A). PC1 did not correlate with HIV infection status, ART status or living in a rural versus urban location; though they did trend positively with living in a rural area, which would be consistent with prior studies showing more *Prevotella*-rich microbiomes in rural versus urban areas in SSA [20]. Both PC2 and PC3 correlated with low alpha diversity (p < 0.001) and higher relative abundance of *Succinivibrio* (Fig. 3A). Individuals in the ART experienced cohort had significantly higher values of PC3 compared to the ART naïve cohort (p < 0.001) and HCs (p < 0.001). Treatment (p-value = 0.039) but not HIV-infection status (p = 0.111) had a significant effect on beta diversity based on Adonis (see Methods Model M5). Living in the rural versus urban location also had a significant effect on beta diversity (Adonis: p = 0.035; see Methods Model M4). PCoA plots stratified by location and week are shown in Figure S10.

We also used beta diversity measures to estimate dysbiosis as the average weighted UniFrac distance between PLWH and HCs (Fig. 3B,C, Figure S11). ART experienced PLWH showed higher dysbiosis compared to ART naïve PLWH (Figure S11), but this was only statistically significant in people recruited in the urban area. However, individuals in the rural area had higher exposure to cotrimoxazole at baseline, and the time of cotrimoxazole at baseline did correlate significantly with dysbiosis (Figure S8), indicating that this may be why change was only observed within the urban cohort. No significant change in dysbiosis over time was found in ART naïve and experienced cohorts, including when stratifying for location (Figure S10). Change in dysbiosis with ART was influenced by baseline dysbiosis (see Methods Model M3), particularly in the rural location (Fig. 3C), with those with the highest dysbiosis at baseline having improvement (negative delta) following 24 weeks of ART/cotrimoxazole treatment and individuals with the lowest dysbiosis at baseline showing worsening dysbiosis (positive delta) at week 24. This same pattern was also statistically significant in the ART experienced cohort though with a smaller effect size (slope) (Fig. 3B), suggesting continuous effects of ART on dysbiosis over time.

### Differential Abundance Analysis

To understand which genera significantly differed between cohorts we used ANCOM-BC2 [44, 45], a differential abundance (DA) analysis package for compositional microbiome data that allows for regression modeling to control for potential confounding factors. Specifically, we determined which taxa differed between HC and the ART experienced or ART naïve cohorts in models that also included the effect of time, viral load, and location to control for confounders. We used both baseline values only (see Methods Model M6) so that we could compare the naïve cohort before ART treatment to the other two cohorts, and a mixed effects model analysis with data from both timepoints (see Methods Model M7), although in the ART Naïve versus HC comparison it is important to note that this includes individuals both before and after ART, and so results will also in part be driven by ART. Analyses were done on bacterial genera classified with the Silva taxonomy [47].

**Figure 4:**
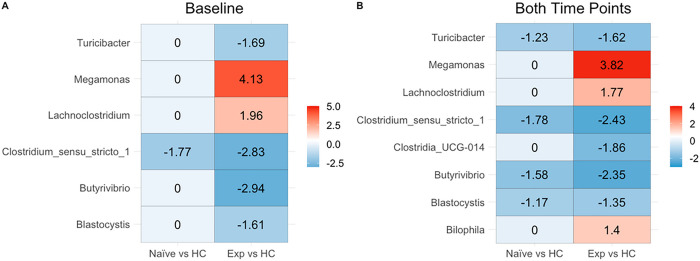
Log-fold changes as compared to ART experienced patients. **(A)** Using ANCOM-BC significant differential log fold changes between microbes were calculated relative to healthy controls using only baseline values. Model used also evaluated the effect of location, viral outcome. A zero means that there is no significant difference in the genus between cohorts. **(B)** Similar to **A,** however both time points were included in the model, therefore time was added to the model, and we controlled for dependence between samples belonging to the same patient.

The ART naïve cohort showed only a significant decrease in the *Clostridium_sensu_stricto_1* genus when compared to HC at baseline. With two timepoints (so also including samples from after 24 weeks of ART), the ART naïve versus HC comparison additionally showed decreases in *Turicibacter, Blastocystis* and *Butyrivibrio* (Fig. 4B). Neither location nor viral load was found to impact DA at baseline.

Comparisons between ART experienced PLWH and HC baseline values showed an increase in *Lachnoclostridium* and *Megamonas* and a decrease in *Turicibacter, Butyrivibrio, Blastocystis* and *Clostridium_sensu_stricto_1* (Fig. 4A). In models created using samples from both time points, a decrease in *Clostridia_UCG-014* in the ART experienced and an increase in *Bilophila* was additionally detected (Fig. 4B). Finally, we also evaluated change over time in the ART naïve cohort (see Methods Model M8) to detect longitudinal microbiome changes with 24 weeks of ART using mixed linear model. Only Genus *Lachnoclostridium* was significant and decreased over time (p-value 7.753354e-05).

### Integrative Analysis of Immune markers and Microbiome

**Figure 5:**
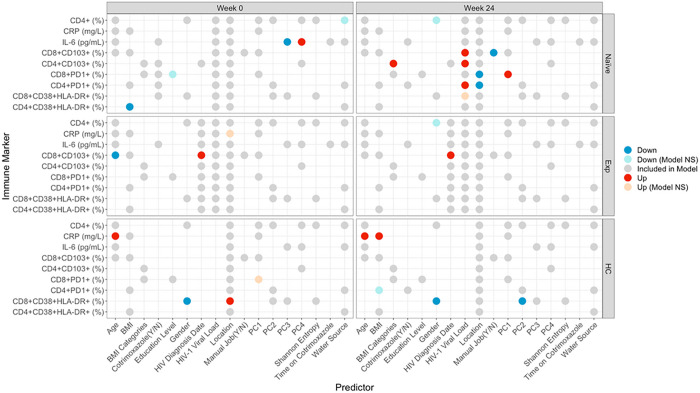
Predictive Models for Immune Markers Each row within each square represents one model. Circles represent predictors included in models. Predictors were determined by backwards stepwise regression feature selection. Location was retained in all models, viral load in the 2 PLWH cohorts, and HIV diagnosis date in the ART experienced cohort. Predictors that had a significant impact on the model are colored red (↑/ positive) or blue (↓/negative). Gender (up) represents higher values in males; location (up) represents higher values in the rural location; BMI categories (up) represents higher values with higher BMI; water source (up) represents higher values in people who drink from wells as opposed to tap; education level (up) represents higher values in people who went to secondary school as compared to tertiary; manual job (up) represents higher values in those who do work manual jobs as compared to those who don’t. “Model NS” means that the overall model was not significant despite the predictor being significant. Grey circles represent non-significant predictors.

To gain further insight into relationships between gut microbiome composition and immune phenotypes, we used linear models with the immune markers described in Fig. 1 as response variables, and measures of microbiome alpha (Shannon entropy) and beta (weighted UniFrac) diversity as explanatory variables. Beta Diversity was summarized as the first 4 PCs in the PCoA analysis described in Fig. 3. We also included several other variables that could potentially influence immune measures in the models including age, BMI/BMI categories, gender, location, HIV diagnosis date (indication of how long PLWH were on ART in the ART experienced cohort) and viral load. We used backwards selection [48] to define a set of predictors that associated with immune markers. Models were customized for each cohort as measurements of viral load would not apply to HCs and the number of years on ART would not influence the ART naïve cohort. Models were then applied to each time point separately (Fig. 5).

For microbiome explanatory variables, we found that in ART naïve individuals only, the inflammatory marker IL-6 was negatively associated with PC3 and positively associated with PC4 of the weighted UniFrac PCoA analysis shown in Fig. 3 (Fig. 5). PC1, whose values correlated with having a more Prevotella-rich/Bacteroides-poor microbiome type, positively correlated with CD8 + PD1 + T cells in the ART naive cohort following 24 weeks of ART treatment (Fig. 5).

Other interesting observations for non-microbiome explanatory variables included that BMI negatively correlated with CD4 + HLADR + CD38 + T cells in ART naïve PLWH only, consistent with a relationship between wasting and disease progression in untreated HIV infection, and positively correlated with CRP in HCs only, which is consistent with previously described relationships between CRP and obesity [49]. Living in the rural versus urban location also influenced immune populations in both the PLWH and HCs. CD4 + and CD8 + T cell exhaustion was higher in the rural location compared to urban after 24-weeks of ART (Fig. 5). CD8 + T cell activation (HLADR + CD38+ ) was also higher in the urban compared to rural area in HC but not HIV-infected cohorts.

Each row within each square represents one model. Circles represent predictors included in models. Predictors were determined by backwards stepwise regression feature selection. Location was retained in all models, viral load in the 2 PLWH cohorts, and HIV diagnosis date in the ART experienced cohort. Predictors that had a significant impact on the model are colored red (↑/ positive) or blue (↓/negative). Gender (up) represents higher values in males; location (up) represents higher values in the rural location; BMI categories (up) represents higher values with higher BMI; water source (up) represents higher values in people who drink from wells as opposed to tap; education level (up) represents higher values in people who went to secondary school as compared to tertiary; manual job (up) represents higher values in those who do work manual jobs as compared to those who don’t. “Model NS” means that the overall model was not significant despite the predictor being significant. Grey circles represent non-significant predictors.

**Figure 6:**
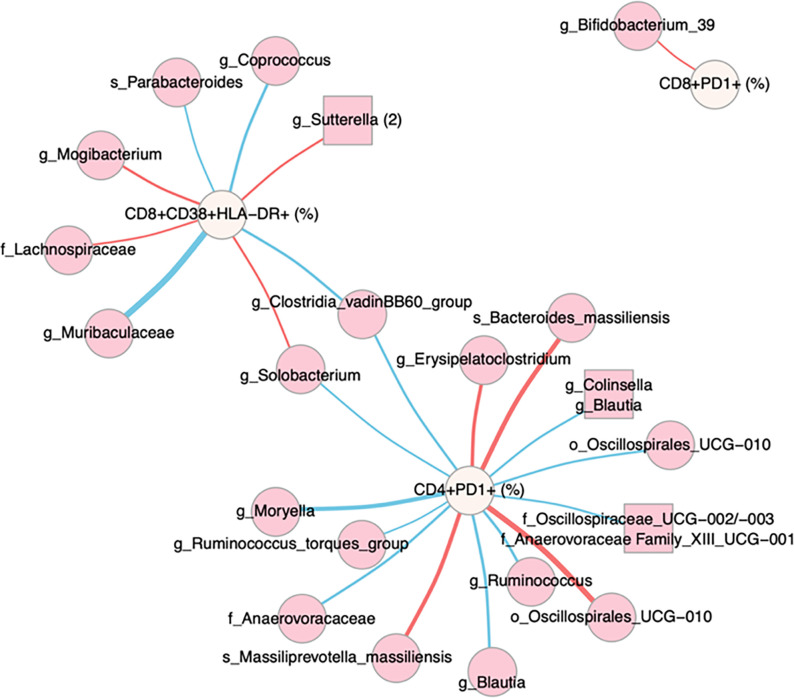
Network analysis of immune and microbial associations Network summarizing relationships between immune markers (beige nodes) and microbial ASVs (dark pink nodes) at Week 0. Modules of co-correlated microbes are represented by squares. Red edges represent positive associations between an immune marker and microbial feature in the ART Naive cohort; blue edges represent negative associations. Edge widths are a function of the p-value on the slope of the ART Naive cohort, with thicker edges representing smaller p-values. Relationships were generated by linear models of the form immune marker ~ microbial feature + microbial feature x cohort, with an additional term for read count of the microbial feature. Relationships in this network are limited to those with an FDR-adjusted p-value on the F statistic of the overall regression < 0.2, adjusted R2 > 0.25, p-value on the slope for the Naïve cohort < 0.05 and different from the slope for the Experienced cohort and/or the healthy controls (p<0.05), and maximum absolute value of DFFITS < 2. Names are based on Silva taxonomy [47] assignment for each ASV. Nodes with more than one listed feature represent highly correlated microbes that were binned using SCNIC [50].

We next used linear regression to identify individual microbes and modules of highly co-correlated microbes (ASVs defined using DADA2 [46], and modules created with SCNIC [50]) associated with immune factors. We performed separate analyses on data from the baseline and week 24 samples. We then formed a network (Fig. 6), where edges represent relationships between an immune population and microbe where the estimated slope for the HIV Naïve cohort was significantly different from either the ART experienced or HC cohort. At baseline, this would identify relationships in untreated infection that are corrected with ART or not present in HCs. Fourteen significant associations were found for CD4 + PD1 + cells, 8 for CD8 + HLADR + CD38 + cells, and 1 for CD8 + PD1 + T cells, and none for the other immune populations tested. Using only baseline data, both negative and positive associations were detected, indicating a potential influence of both protective and detrimental bacteria. When performing the same analysis using only the data from the sampled collected at the 24 weeks timepoint, only 2 associations were found, showing a diminishing of these relationships with effective ART.

To evaluate microbe-immune relationships specific to treated infection versus HCs, we used a similar approach to identify relationships between ASVs and immune phenotypes that were significant in ART experienced PLWH but not HCs (see Methods Model M9). These tests showed a much weaker effect, identifying 2 associations with CD4 + PD1 + T cells at week 24 (Specifically, a positive relationship with an ASV in the family *Enterobacteriaceae* (p-value of slope in Exp = 4.2 e^− 4^) and a negative relationship with an ASV in the order *Oscillospirales*: UCG-010 (p-value of slope in Exp = 3.3e^− 3^), a positive relationship between an ASV in the genus *Gastranaerophiliales* with CD4 + PD1 + T cells at baseline (p-value of slope in Exp = 1.4 e^− 6^) and a negative association between an ASV in the *Eubacterium siraeum* group with CD4 + CD103 + T cells at baseline (p-value of slope in Exp = 6.1 e^− 9^).

## Discussion

Although substantial gains in ART coverage for PLWH in SSA have led to improvements in life expectancy and reduced mortality [51], adherence and access is challenging, particularly in rural areas [22]. In this study, all participants with HIV-1 were treated with EFV/3TC/TDF [52]. Of the individuals on EFV/3TC/TDF at baseline, 82.1% had viral control and of those who were ART naïve at baseline, 77.6% achieved viral control after 24 weeks of therapy. These levels are consistent with previous reports of an 81% viral control rate with EFV/TDF/FTC [52], with failures related to both discontinuation due to adverse events and the development of antiviral resistance. In contrast to prior studies describing increased failure of first-line ART in rural versus urban South Africa [21], we did not find a significant difference in the percent of individuals who achieved viral control with ART among individuals recruited in a large polyclinic located in an urban area versus a district hospital serving people from surrounding rural communities.

As expected, ART naïve PLWH had lower CD4 + T cell percentages and CD4+/CD8 + T cell ratios. We observed significantly higher CD4 + T cell percent and CD4/CD8 T cell ratio in ART naive women compared to men, which is consistent with the results of a prior study conducted in Nigeria [53]. ART naïve PLWH had significantly elevated CD4 + and CD8 + T cell activation (CD38 + HLA-DR+) and exhaustion (PD1+), and increased levels of the pro-inflammatory cytokine IL-6 compared to healthy controls, as is well known to occur [54–57]. When using linear models to test whether demographic variables (age, BMI, Gender), living in the rural versus urban area, HIV viral load or diagnosis date, or composition of the gut microbiome had a significant influence on these values in the ART Naïve cohort at baseline, only a couple of significant relationships were identified (Fig. 5). First, levels of activated CD4 + CD38 + HLA-DR + T cells negatively correlated with BMI, consistent with this immune marker and wasting occurring with progression to AIDS [58]. Second, the inflammatory marker IL-6 was negatively associated with PC3 and positively associated with PC4 of the weighted UniFrac PCoA analysis. A negative correlation between IL-6 and PC3 is interesting, because individuals in the ART experienced cohort had significantly higher values of PC3 compared to the ART naïve cohort (p < 0.001), suggesting that ART may be altering the microbiome to a less-inflammatory state, even though it is a state with lower alpha diversity.

When restricting our subsequent analyses to only individuals who achieved viremic control, we observed significant reductions in levels of chronic T cell activation and exhaustion with ART. Interestingly, in stratified analyses, we found that these reductions were mostly significant only when considering individuals from the urban and not the rural location. This was the case both when comparing individuals who were ART naïve versus experienced at baseline and when looking at longitudinal changes that occurred before and after 24 weeks of ART. Prior studies have described significant challenges in administering ART in rural areas of SSA, such as longer travel distances to access health care facilities, increased stigma, and human resources challenges [21, 22, 59]. In one study conducted in rural and urban areas of South Africa, individuals in the rural group were more likely to have failed first-line ART treatment for longer and to have drug resistance mutations [21]. However, we observed this lack of improvement in immune activation, exhaustion, and inflammatory markers in the rural area despite a lack of difference in rate of treatment failures and in a cohort restricted to just those with suppressed virus, suggesting factors besides access to care are likely to be important. In one prior study conducted in Uganda, high levels of inflammatory markers (IL-6 and d-dimer) among PLWH on ART were linked with economic insecurity, Specifically a lack of electricity and an unprotected water source [60]. Further work is needed to determine whether such factors may underlie the differences that we observed between rural and urban immunologic ART response.

One surprising result of our study was that individuals in our HC cohort and the cohort of people on effective ART at both timepoints had some worsening of immune phenotypes over time. For instance, the HC had a significant increase in CD8 + PD1 + T cells. Additionally, when stratified by location, the ART experienced cohort in the rural location had an increase in the inflammatory marker CRP over time (Fig. 1). One potential driver of increased immune exhaustion and inflammation in the HC and ART experienced cohorts respectively, was that in the time during which patient samples were collected, January 2018 – August 2019, there was socio-economic change in Zimbabwe. One indicator of this is increases in inflation rates, which ranged from 3.52–66.8% during baseline sample collections (which were from January 2018 to March 2019) and from 4.29–230.54% during 24 week sample collections (which were from July 2018 to August 2019) [40].

Since one goal of this study was to relate immune markers in HIV to the intestinal microbiome, we were also interested in evaluating T cells expressing the mucosal trafficking marker CD103. The chief ligand for CD103 is E-cadherin, a cellular adhesion molecule found on epithelial cells which is important for T cell homing to mucosal sites, including the intestine [61]. Circulating CD103 + T cells share a cellular transcriptome that more closely resembles CD4 + T cells from the gut, suggesting they are homing to or from the gut [62]. Mucosal trafficking (CD103 percent) was highest in the ART experienced cohort and lowest in the ART naïve cohort and also increased with treatment in longitudinal analyses. The decrease in ART naïve PLWH compared to HCs could indicate trafficking to mucosal sites. The increase with ART could be indicative of a reduction in gut homing of T cells due to a reduction of mucosal inflammation within the intestinal compartment.

Our study population overall had a relatively Prevotella-rich/Bacteroides-poor microbiome, which is consistent with other studies conducted in SSA [16, 63, 64]. Individuals in the rural area had a significantly different microbiome composition compared to urban, as has been previously observed in Cameroon and Tanzania [19, 63]. This was not driven solely by differences in the PC axis that separated Prevotella-rich from Bacteroides-rich microbiomes, which has previously been described to represent a “Westernization” of the microbiome with urbanization [63]. Interestingly, PC1 positively correlated with CD8 + PD + 1 T cell exhaustion in the ART naïve cohort after 24 weeks of treatment. This may be related to the findings that individuals in the rural area trended towards higher PC1 values and did not respond as well to ART as urban, and thus had elevated CD8 + PD1 + T cells remaining with ART.

Overall, differences with untreated HIV infection were not pronounced, as has been indicated previously [17]. HIV infection status did not have a significant effect on beta diversity independent of treatment, and following differential abundance analysis, only 1 ASV in Clostridium cluster I had a significantly reduced abundance in ART naïve PLWH compared to HCs. However, the gut microbiome of ART naïve PLWH did have reduced alpha diversity compared to HCs, but this was only observed in the rural and not the urban location. A reduction in alpha diversity in ART naïve PLWH compared to HCs has been reported previously in SSA [14] and Western countries [65], but inconsistently [17], and linked with disease severity [14]. A potential confounder in our study is that some ART naïve PLWH were taking the antibiotic cotrimoxazole at baseline, more commonly in the rural site. However, this is likely not the driving factor of the reduced diversity observed since time on cotrimoxazole did not correlate with baseline alpha diversity values. Furthermore, cotrimoxazole was also not found to decrease alpha diversity in randomized control trials of HIV-exposed infants and preschool children in studies conducted in SSA [66, 67]. Although lower alpha diversity has been linked with disease severity in prior studies of ART naïve PLWH in SSA[14], the observation of lower alpha diversity in ART Naïve PLWH compared to HCs in rural areas also does not appear to be related to more severe disease since the ART naïve individuals in the rural area did not have significantly lower CD4 + T cell percent or CD4/CD8 T cell ratio or higher viral loads at baseline.

Despite a lack of pronounced microbiome differences in ART naïve PLWH, we did find significant relationships between gut microbiome composition and inflammation. Specifically, an association between PC3 and PC4 of the weighted UniFrac PCoA analysis and levels of IL-6 was observed in the ART naïve cohort in linear models. Furthermore, when using pairwise linear regression and network analysis to identify ASVs that were correlated with immune phenotypes Specifically in the ART naïve PLWH, CD4 + PD1 + was the strongest hub, followed by CD8 + CD38 + HLA-DR + T cells. ASVs that were positively correlated with these cell populations included bacteria that have been associated with bacteremia, intra-abdominal infections, colorectal cancer and/or inflammatory bowel diseases including *Bacteroides massiliensis* [68], *Prevotella massiliensis* [69], *Erysipelatoclostridium*, a genus containing *Thomasclavelia ramosa* (formally *Clostridium/Erysipelatoclostridium ramosum*) [70], and Sutterella [71, 72]. *Bacteroides massiliensis* is additionally a mucin degrader with a strong preference for host mucin glycans over dietary substrates [73], an activity that has been shown to thin the mucous layer essential for maintaining barrier function in cancer models [74]. *Prevotella massiliensis* has previously been found to correlate with CD8 + CD38 + HLA-DR + cells in ART experienced HIV positive MSM [17]. Many of the bacteria that negatively correlated with these cell populations were poorly defined or had unclear effects on health, but included bacteria previously associated with disease protection such as *Parabacteroides* [75], and *Coprococcus* [76]. *Coprococcus* has been associated with improved barrier function among ART experience HIV positive MSM previously [77] and is known to produce the beneficial metabolite butyrate that can protect barrier function [78]. Taken together, these results suggest that microbiome composition in ART naïve PLWH may be important for influencing levels of immune activation and exhaustion driven by translocation. Although we do not have laboratory validation supporting causality in this case, prior work in our lab showed that the fecal bacteria from ART naïve PLWH in the United States had a particularly strong ability to stimulate CD8 + CD38 + HLA-DR + T cells in *in vitro* stimulations of Peripheral Blood Mononuclear Cells (PBMCs) despite a lack of pronounced microbiome differences compared to HCs [10]. These levels of *in vitro* stimulation correlated with *ex vivo* levels in matched patient blood, supporting clinical significance [10].

The ART experienced cohort had more pronounced microbiome differences from the HCs compared to ART naïve PLWH. ART was associated with lower alpha diversity in both cross-sectional comparisons and longitudinal analysis of the rural site. ART status also had a significant relationship with beta diversity, and more taxa significantly differed between the ART experienced cohorts and HCs than between ART naïve and HCs. Since ART treatment was coupled with use of the prophylactic antibiotic cotrimoxazole, it is difficult to disentangle the effects of ART drugs from the effects of the antibiotic. Our results suggest some influence of cotrimoxazole, since dysbiosis (but not alpha diversity) correlated positively with length on cotrimoxazole in the ART naïve PLWH at baseline. This is consistent with cotrimoxazole being associated with changes in composition but not a loss of alpha diversity in a placebo-controlled trial [66]. In another study where children in Zimbabwe and Uganda were randomized to either temporarily suspend prophylactic cotrimoxazole use or not [79], cotrimoxazole suspension resulted in increases in certain types of Streptococcus and increased inflammation, but not global differences in gut microbiome composition.

Reduced alpha diversity with ART plus cotrimoxazole compared to ART naïve PLWH is consistent with other studies evaluating PLWH exposed to both ART and cotrimoxazole [12] and to PLWH exposed to ART only [80–83]; although increased diversity with ART has also been observed [65] and related to longer duration of ART [13]. Even within this study, whether alpha diversity was gained or lost with ART depended on baseline values, suggesting differential effects based on the level of HIV-associated disturbances at the commencement of treatment. Whereas a previous study in Cameroon found a more pronounced effect of ART regimens containing ritonavir-boosted protease inhibitor (PI/r)-based ART compared to NNRTI based regimes [12], one study in Mexico showed reduced diversity with both the same ART regimen used here and PI/r-based ART [83]. Taken together, there is more literature support for an effect of ART compared to cotrimoxazole on gut alpha diversity.

Taxa that were significantly enriched in ART experienced PLWH compared to HC include *Megamonas, Bilophila*, and *Lachnoclostridium*, which have all been found to be increased in various prior studies of microbiome differences with treated HIV [8, 15, 84]; *Megamonas* has additionally been associated with iron deficiency in the context of treated HIV infection in resource limited settings [15], and both *Bilophila* and *Megamonas* can be pro-inflammatory [15, 85]. Taxa that were depleted in the ART experienced PLWH included *Turicibacter, Butyrivibrio, Blastocystis*, and other poorly defined ASVs in the order Clostridiales. Despite the possibility for microbiome differences that are observed with ART to be involved in inflammation, we found fewer correlations between immune phenotypes and the microbiome within ART experienced PLWH. Linear models indicated that microbiome differences not correlated with ART, namely microbiomes that differentiated by Prevotella versus Bacteroides ASVs (PC1 axis), were related to levels of immune exhaustion in treated infection. Linear models used to identify relationships between individual microbes and immune populations in ART experienced PLWH identified only a few correlations, again with immune exhaustion. These included associations between CD4 + PD1 + T cells and Gastranaerophilales (positive), Oscillospirales (negative), and Enterobacteriaceae (positive).

Our study does have some weaknesses. Since we only used 16S rRNA targeted sequencing of bacteria to assess microbiome composition, our results do not rule out a role for fungi, parasites, viruses, strain level variation or differences in expressed functions. Furthermore, although prior work in our laboratory has been able to validate immune modulation by fecal bacterial communities using in vitro assays or gnotobiotic mice [56], we collected fecal samples in a preservative for DNA integrity but not amenable to such functional experiments because of challenges with getting samples from rural Zimbabwe to Colorado, USA. Finally, we were surprised to find differences over time in immune readouts our non-intervention cohorts, and although we suspect that changes in economic stability that occurred over the time of sampling may have been at play, we did not measure factors such as changes in access to food, clean water, or stress over time to confirm drivers.

## Conclusion

We found that although the percent of individuals who achieved viral control with ART was not different between PLWH in rural versus urban Zimbabwe, immunologic improvements with successful ART were muted in the rural compared to the urban area for inflammatory, T cell activation and T cell exhaustion phenotypes. More work is needed to determine underlying factors that may drive these differences. We found that ART with cotrimoxazole prophylaxis had a relatively strong impact on gut microbiome composition compared to HIV infection alone, with reductions in alpha diversity and greater deviation from the microbiome composition of HCs. Furthermore, we observed significant relationships between gut microbiome composition and inflammation, T cell exhaustion and chronic immune activation markers particularly in ART naïve PLWH, but also in the context of treated infection.

## Methods

### Recruitment

The rural recruitment site was the Mutoko District Hospital, which is in a small town (population of about 12,500) about a 2 hour drive from the city of Harare, that services surrounding rural villages. The urban subjects were recruited from the Mabvuku Polyclinic, a large urban clinic administered by the City of Harare. Subjects were excluded from all cohorts if they had used antibiotics (apart from co-trimoxazole) within the prior two months, were pregnant, or had a Body Mass Index (BMI) greater than 29 kg/m2(are obese). All participants were 18 years old or older.

### Fecal and Blood Specimen Collection

At the first visit after informed consent was obtained, study participants were given a fecal collection kit. Stool samples were collected in a specimen collector within 24 hours prior to their second and third clinic visits, and aliquoted by the study participant into an OmniGene Gut collection system (OM-200, DNA Genotek, Ontario, Canada) for preservation of DNA. A fasting blood sample was collected by venipuncture during the second and third visits. Blood and fecal samples from the rural clinic were couriered to the Infectious Diseases Research Laboratory in the Internal Medicine Unit at the University of Zimbabwe Faculty of Medicine and Health Sciences (Harare), which is a 2 hour drive from the Mutoko District Hospital, in a cooler on the day of sample collection and then stored at −80°C. Samples for microbiome sequencing were shipped on dry ice to the University of Colorado Anschutz Medical Campus (Aurora, CO) and stored at −80°C upon arrival.

### Collection of Demographic Information

Study participants filled out a questionnaire which included questions on age, Body Mass Index (BMI), gender, education level, water source, and factors related to occupation and home life.

### Blood Sample Processing

A subset of the blood sample was analyzed by flow cytometry at the Infectious Diseases Research Laboratory (IDRL) located in the Internal Medicine Unit at the University of Zimbabwe Faculty of Medicine and Health Sciences. Whole blood was collected in BD Vacutainer tubes containing sodium heparin and red blood cells (RBCs) from 500 μL of blood were lysed with 1x RBC lysis Buffer (Thermo Fisher). Cells were washed twice with staining buffer containing PBS, 2% BSA, 1mM EDTA and surface stained with BV785-labelled anti-CD3 antibody (BioLegend Cat# 317330), PerCP/Cy5.5-labelled anti-CD4 (BioLegend Cat# 317428), BV421-labelled anti-CD8 antibody (BioLegend Cat# 344748), BV605 labelled anti-CD38 antibody (BioLegend Cat# 303533), Pe-labelled anti-CD103 antibody (BioLegend Cat# 350206), Pe-Cy7 labelled anti-HLA-DR antibody (BioLegend Cat# 307616) and FITC-labelled anti-PD-1 antibody (BioLegend Cat# 329904) or appropriate fluorescence minus one (FMO) controls. Cells were washed twice with staining buffer and fixed in 1% formaldehyde. Cells were acquired on a BD LSRFortessa Flow Cytometer and analyzed by FlowJo.

Plasma was isolated and frozen for shipment to the University of Colorado for measurement of CRP and IL-6 with ELISA following the manufacturer’s protocol (CRP: R&D Systems cat. DCRP00, IL-6 Invitrogen cat. 88–7066). Blood samples were also used to evaluate absolute CD4 + T cell count or CD4 + T cell percent using the Sysmex (formally Partec) CyFLOW Cytometer, and CD4 easy count kit or CD4 percent easy count kit following manufacturer’s instructions (Sysmex, cat: 058401, 058505, respectively), and HIV viral load using a Roche COBAS AmpliPrep/COBAS TaqMan (CAP/CTM) instrument with COBAS AmpliPrep/TaqMan HIV-1 test v2.0 kit following manufacturer’s instructions at the Infectious Diseases Research Laboratory (IDRL) located in the Internal Medicine Unit, UZ Faculty of Medicine and Health Sciences (UZFMHS).

### DNA Extraction and Sequencing

DNA was extracted using the DNeasy PowerSoil Kit protocol (Qiagen). Extracted DNA was PCR amplified with barcoded primers targeting the V4 region of 16S rRNA gene according to the Earth Microbiome Project 16S Illumina Amplicon protocol with the 515F:806R primer constructs [86]. A sterile water blank was included in each batch of extractions and PCR amplification to serve as a procedural control. Each PCR product was quantified using PicoGreen (Invitrogen), and equal amounts (ng) of DNA from each sample were pooled and cleaned using the UltraClean PCR Clean-Up Kit (MoBio). Sequences were generated on two runs on a MiSeq personal sequencer (Illumina, San Diego, CA).

### Software

Unless otherwise specified, all analyses were run in R version 4.2.2 (2022–10-31) [87].

### Data Preprocessing

Before analyses were performed one sample with no immune data (ZIM033.3) was removed. ART naïve patients at week 24 and ART experienced patients at week 0 were labelled as “viremic” if they had more than 200 copies of HIV/mL of blood [26]. There were 10 patients whose CD4 T cell immune data was not able to be measured. Therefore, we imputed values for that missing data, Specifically, for the following immune markers: CD4 + CD38 + HLADR+ (%), CD4 + PD1+ (%), CD4 + CD103+ (%). Imputed values were calculated as the average immune marker value. Length on ART was calculated for the ART experienced PLWH as the number of days between the visit date and the date that they started ART. For all longitudinal analyses only study participants who provided samples at both baseline and week 24 were included.

### Immune Phenotype Analyses

Fixed-effects Ordinary Least Squares (OLS) Linear Modeling

Linear modeling performed in Figure S3 (M1) used the *feols* function in the *fixest* package [88] in R to run fixed-effects OLS linear modeling.

(M1) Immunemarker~Cohort+Week+Cohort*Week

Model (M1) was run on all study participants and then stratified by location and clustered by Person ID (PID), due to the longitudinal aspect of the study. *ggplot* [89] was then used to visualize results. Immune marker values were not imputed for this analysis.

### Inter and Intra Cohort Immune Analyses

For the inter-cohort tests (Fig. 1), all baseline values were used regardless of whether the study participant completed the 24 week visit. ART experienced individuals who were viremic at baseline were excluded to evaluate the effects of successful ART. Significant differences were assessed with a Kruskall-wallis test with a Dunns post hoc. In the intra-cohort longitudinal comparisons, study participants who did not complete both the baseline and 24 week visit were excluded as were all viremic patients (including ART naïve and experienced). Significant differences were assessed with a paired Mann Whitney U test. No immune marker means were imputed in this analysis. *ggplot* [89] was used to visualize results.

### Microbiome Analyses

#### Core Metrics Analysis and Taxonomic Classification

Demultiplexing of 16S rDNA gene sequences and quality control using DADA2 [46] to define ASVs were performed in QIIME2 (version 2023.5) [90]. SILVA (version 138) [91, 92] was used to perform taxonomic classification of each ASV. Taxa that were not classified at the phylum level or that were classified as mitochondria and chloroplasts were excluded. SEPP [93] was used to produce a phylogeny of ASVs for use in Diversity analyses. The feature table of ASVs was rarified to a sampling depth of 16,645 sequences per sample prior to downstream analyses.

Alpha diversity, measured as Shannon entropy [41], was plotted across all three cohort and over time (Fig. 2A,B) using QIIME 2. Linear modeling (Fig. 2C) (M2) was performed using the *lm* function in stats package [87].

(M2) ΔShannonEntropy~BaselineShannonEntropy

Beta diversity, measured using weighted UniFrac distances [43], was calculated using the core metrics functions in QIIME2. Dysbiosis was calculated in R as the mean distance of each sample in the ART naïve and experienced cohort to all healthy controls of the same time point. Linear modeling (M3) was then performed using the *lm* function in stats package [87] for each cohort and each location separately (Fig. 3B,C).

(M3) ΔDysbiosis~BaselineDysbiosis

Weighted UniFrac PCoA results from the core metrics analysis were used to calculate coordinates for biplots in QIIME2 and then visualized in R. To evaluate potential moderators of beta diversity two different Adonis tests [94] were performed in R. The models that were run were:

(M4) WeightedUniFrac~Location+Week+Location*Week

(M5) WeightedUniFrac~Age+HIVStatus+TreatmentStatus

Both models M4 and M5 were stratified (using the strata parameter) by PID to account for the dependence between samples from the same patient. Age was included in model M5 as a potential confounder since it significantly differed across cohorts.

### Compositional Differences

To determine genera that significantly differed in abundance across cohorts, we used ANCOM-BC (version 2.0.2) [44]. This allowed us to account for the compositional nature of microbiome data, control for location and viral control with ART as potential confounders, and account for the longitudinal study design. Analysis was performed in R using the Phyloseq (version 1.42.0) [95], and Qiime2R (version 0.99.6)[96] packages. The taxonomic level that we evaluated at was genus and the reference group used in the analysis were the healthy controls. Subsequently, three models were tested.

(M6) FilteredTaxa~Location+ViralOutcome+Cohort

Model M6 was run only on baseline values. Also, values were grouped by cohort which is used to help detect structural zeros and performing multi-group comparisons. Results are shown in Fig. 4A.

(M7) FilteredTaxa~Location+Week+ViralOutcome+Cohort+(1∣PID)

Model M7 evaluated values at both time points (both week 0 and week 24) and grouped by cohort as in model M6. The random effects term was added to account for the dependence between samples of the same patient. Results are shown in Fig. 4B, and it should be noted that since the second timepoint was after 24 weeks of ART/cotrimoxozole these additional differences from baseline also will reflect changes with ART.

(M8) FilteredTaxa~Location+Week+ViralOutcome+(1∣PID)

Model M8 evaluated values at both time points but only for those patients who were ART naïve PLWH at baseline, to evaluate changes over time with ART. No results were visualized for this model as there were very few differences. For models M6 and M7, visualizations (Fig. 4A,B) were produced using *ggplot* [89] and *microViz* [97].

### Integrative Analysis of Immune Markers and Microbiome

#### Predictive Models for Immune Markers

To understand the impact that other variables might have on patient immunity (Fig. 5) we curated a list of potential clinical and demographic features: Cohort, location, gender, BMI, BMI categories, HIV status, week, age, Bristol stool score, education level, water source, cotrimoxazole, length on cotrimoxazole (months), normal work transportation, manual work, manual chores at home, head of household, and length on ART (years); and measures of microbial community diversity (PCA1, PCA2, PCA3, PCA4 from the weighted UniFrac PCoA and Shannon entropy). Viral load was included in the models pertaining to PLWH (naïve and experienced). HIV diagnosis date was included in models pertaining only to ART experienced PLWH. Since linear models inherently penalize large numbers of explanatory variables [98] because more variables in the model can reduce results accuracy due to overfitting, we reduced the number of explanatory variables when developing the models using backwards stepwise regression feature selection [48]. Models were created for each immune marker, cohort and time separately using the *lm* function from stats package [87].

#### Network analysis of immune and microbial associations

Data for 8 immune markers and 237 microbial features were combined in a dataset spanning 127 participants at baseline and 113 participants at Week 24. Analyses were run separately for baseline and week 24 data. Microbial features were limited to those observed in > 20% of samples, resulting in 191 single ASVs and 46 modules of highly-co-correlated ASVs aggregated by SCNIC using default parameters [50]. Features were expressed as relative abundance. For each timepoint, linear regressions were performed for each pair of immune markers and microbial features as previously described [99]. The form of the model was:

(M9) ImmuneMarker~MicrobialReadCount+MicrobialFeature+MicrobialFeature*Cohort

The read count term accounted for differences in across samples since this analysis did not use rarefied data. To identify relationships that were evident in ART naïve PLWH and different from ART experienced PLWH or HCs, resulting models were filtered to include only those with an FDR-adjusted p-value on the F statistic of the overall regression < 0.2, adjusted R2 > 0.25, p-value on the slope for the ART naïve cohort < 0.05 and different from the slope for the ART experienced cohort and/or the HCs (p < 0.05 for at least one of these slopes), and maximum absolute value of DFFITS < 2 (to exclude results that were outlier driven). The resulting network and models were visualized using the VOLARE [99] web application. A high-resolution version of the network was generated with the *igraph* [100] library in R. To compare microbe:immune relationships observed in ART experienced PLWH to HCs, data was filtered to exclude the ART naïve cohort. Resulting models were limited to those with FDR-adjusted p-value on the F statistic of the overall regression < 0.2, adjusted R2 > 0.25, one of the slopes for the 2 cohorts different than 0 and the slopes for the 2 cohorts being different than each other ((pSlopeExp < 0.05 | pSlopeHC < 0.05) & pExp_v_HC < 0.05), maximum absolute value of DFFITS < 2.

## Figures and Tables

**Table 1:** Summary of demographics

	Overall	ART Naïve PLWH (Naïve)	ART Experienced PLWH (Exp)	Healthy controls (HC)	p-value		
					Naïve vs Exp	Naïve vs HC	Exp vs HC
		
	**(N=148)**	**(N=67)**	**(N=39)**	**(N=42)**			
**Arm**							
Rural	75 (50.7%)	34 (50.7%)	19 (48.7%)	22 (52.4%)	NS	NS	NS
Urban	73 (49.3%)	33 (49.3%)	20 (51.3%)	20 (47.6%)			
**Sex**							
Female	85 (57.4%)	39 (58.2%)	23 (59.0%)	23 (54.8%)	NS	NS	NS
Male	63 (42.6%)	28 (41.8%)	16 (41.0%)	19 (45.2%)			
**Age (years)**	37 [29–44]	35 [27–39]	45 [42–51]	36 [28–41]	***	NS	***
**BMI (kg/m^2^)**	22 [20–24]	23 [20–24]	22 [19–24]	23 [21–26]	NS	NS	*
**BMI Cathegory**							
Severe Thinness <16.0	3 (2.03%)	1 (1.49%)	2 (5.13%)	0			
Moderate Thinness <17.0	1 (0.68%)	0	0	1 (2.38%)			
Mild Thinness <18.5	9 (6.08%)	3 (4.48%)	5 (12.8%)	1 (2.38%)	NS	NS	NS
Normal 18.5–24.9	105 (70.9%)	51 (76.1%)	26 (66.7%)	28 (66.7%)			
Overweight ≥25.0	25 (16.9%)	10 (14.9%)	5 (12.8%)	10 (23.8%)			
Obese ≥30.0	5 (3.38%)	2 (2.99%)	1 (2.56%)	2 (4.76%)			
**CD4 T cells count (cells/μL)**	300 [150–430]	260 [120–350]	390 [230–550]	NA [NA-NA]	**	NA	NA
**CD4 T cells (%)**	36 [19–49]	22 [12–36]	36 [20–45]	56 [48–63]	**	***	***
**CD4/CD8 T cell ratio**	0.70 [0.32–1.2]	0.39 [0.18–0.68]	0.67 [0.34–0.97]	1.7 [1.1–2.1]	**	***	***
**ART exposure (months)**	NA [NA-NA]	0	89 [69–114]	NA [NA-NA]	NA	NA	NA
**Co-trimoxazole exposure (months)**	0 [0–14]	0.030 [0–0.22]	84 [60–99]	0 [0–0]	***	***	***
**Viral Load (copies/mL)**	4194 [19–48992]	26491 [3824–71217]	0 [0–19]	NA [NA-NA]	****	NA	NA
**Viral Load Outcome**							
Virologic failure	21 (14.2%)	15 (22.4%)	6 (15.4%)	0 (0%)	NS	***	**
**Both Visits**							
Yes	133 (89.9%)	59 (88.1%)	37 (94.9%)	37 (88.1%)	NS	NS	NS
No	15 (10.1%)	8 (11.9%)	2 (5.1%)	5 (11.9%)			
